# Psychological burden of achalasia: Patients’ screening rates of depression and anxiety and sex differences

**DOI:** 10.1371/journal.pone.0285684

**Published:** 2023-05-11

**Authors:** Franz Hanschmidt, Julia Treml, Julia Deller, Nicole Kreuser, Ines Gockel, Anette Kersting

**Affiliations:** 1 Department of Psychosomatic Medicine and Psychotherapy, University Hospital Leipzig, Leipzig, Germany; 2 Department of Visceral, Transplant, Thoracic and Vascular Surgery, University Hospital Leipzig, Leipzig, Germany; University of Southern Denmark, DENMARK

## Abstract

**Objective:**

Achalasia is associated with reduced quality of life in affected patients but research regarding the psychological burden of achalasia in terms of depression and anxiety is scarce. The current study therefore aims to investigate rates of depression and anxiety in patients with achalasia in relation to prevalence rates in the general population and to examine the extent to which achalasia-related characteristics (time since diagnosis, symptom load, achalasia-related quality of life, treatment history) predict symptoms of depression and anxiety.

**Methods:**

Using validated screening instruments, rates of depression and anxiety were assessed in a cross-sectional survey of a sample of 993 patients with achalasia and compared to population controls stratified by age and sex. Associations between depression and anxiety and achalasia-related factors were explored using linear regression.

**Results:**

Compared to population controls, screening rates of female patients with achalasia were between 3.04 (p = .004) and 7.87 (p < .001) times higher for depression and 3.10 (p < .001) times higher for anxiety, respectively. No significant differences were found for male patients with achalasia. Both achalasia-related quality of life and symptom load were independently related to impaired mental health.

**Conclusion:**

Women appear to be specifically affected by the psychological burden of achalasia, pointing to sex-specific or gendered experiences of the disease. In addition to symptom reduction, psychological support may prove beneficial for improving the well-being of patients with achalasia.

## Introduction

Achalasia is a rare esophageal disease characterized by the absence of peristalsis in the esophageal body and impaired relaxation of the lower esophageal sphincter [1]. Common symptoms of achalasia include dysphagia, regurgitation, heartburn or chest pain, and in more advanced stages, loss of weight and malnutrition [1, 2]. Studies have reported prevalence rates of achalasia between 15.3 and 27.1 per 100,000 persons [3, 4]. The underlying causes of achalasia remain unknown, and only palliative treatment options aimed at reducing symptoms are available, with symptoms persisting or re-occurring in up to 20% of patients after therapeutic intervention [1, 5]. Thus patients with achalasia may experience significant long-term reductions in disease-specific and general quality of life [6–8].

A number of factors may increase the risk for mental health problems in patients with achalasia. Individuals living with a chronic medical condition such as achalasia have been shown to be more likely to develop depression or anxiety [9, 10]. In addition to the psychological burden resulting from symptom load, social and functional impairments may contribute to depressive and anxiety symptoms. Patients with achalasia have reported that their condition conflicted with their social activities, interpersonal relationships, and leisure activities [11]. In a study by Ekberg et al., 41% of patients with dysphagia described experiences of panic and anxiety during meals, and over one-third (36%) stated that they avoided eating in the presence of others [12]. Despite the indication of increased psychological burden, information on the prevalence of specific mental health disorders, such as depression and anxiety, in patients with achalasia are scarce. A recent study demonstrated that patients with achalasia are more likely to develop depressive symptoms within one year of diagnosis than patients without achalasia [13]. Other available studies have assessed patients’ general mental health status in terms of disease-specific and general quality of life, and few studies have included appropriate population controls [7, 8]. These studies suggest that patients with achalasia who do not respond to therapeutic treatment have worse mental health than the general population, whereas patients who showed reduced symptom load after therapeutic treatment did not report impaired mental health [7]. However, lacking assessment of disorder-specific mental health outcomes such as depression or anxiety limits the clinical relevance of findings and healthcare providers’ ability to provide targeted intervention recommendations.

This study sought to investigate rates of depression and anxiety in patients with achalasia in relation to prevalence rates in the general population. Another aim was to investigate the extent to which achalasia-related characteristics (time since diagnosis, symptom load, achalasia-related quality of life, treatment history) predict depressive and anxiety symptoms.

## Methods

### Design and participants

All data were collected in a cross-sectional study using self-report questionnaires. Participants were recruited by searching the patient registry established as part of the Achalasia Risk Consortium, a European-wide study aiming at identifying genetic causes of achalasia (https://www.achalasie-konsortium.de/). The following inclusion criteria were applied to identify patients eligible for this study: 1) German-speaking; 2) Minimum age of 18 years; 3) Confirmed diagnosis of achalasia; 4) No other rare esophageal motility dysfunction. Between 04/2016 and 12/2018, a total of 1801 eligible patients were contacted by postal mail or email and invited to participate in the study. All eligible patients had the option to complete the questionnaire using a paper-pencil version or online survey via link. The invitation letter included detailed information on the study, an informed consent sheet, the study questionnaire, and a pre-paid return envelope. The online survey was administered via Unipark Questback EFS (unipark.com). The study was conducted according to the ethical principles laid out in the Declaration of Helsinki and was approved by the Ethics Committee of the University of Leipzig.

### Materials

Participants’ socio-demographic characteristics, general health status (current comorbidities, lifetime help-seeking for mental problems), and several aspects of achalasia-related characteristics (time since diagnosis, treatment history) were assessed with self-generated questions.

The German versions of the Patient Health Questionnaire-9 (PHQ-9) and the Generalized-Anxiety-Disorder-7 (GAD-7) were used to screen for depressive and anxiety symptoms, respectively [14, 15]. The PHQ-9 consists of nine items measuring the severity of symptoms of major depressive disorder. The GAD-7 contains seven items assessing the severity of symptoms of generalized anxiety disorder. All items are scored on a scale from 0–3 and summed to obtain a total score (PHQ-9 range: 0–27, GAD-7 range: 0–21). For both scales, a cut-off score of ≥ 10 has proven effective in identifying individuals with depression and generalized anxiety disorder, respectively [13, 14]. Reliability and validity of both scales have been shown (Cronbach’s alpha in this study: .86 [PHQ-9]; .90 [GAD-7]) [14, 15].

Achalasia symptom severity was measured with the Eckardt score [16]. The Eckardt score queries four symptoms, including dysphagia, chest pain, regurgitation, and weight loss. Each symptom is scored on a scale from 0 to 3, with higher values indicating higher symptom severity. A total score is calculated by summing the individual item scores (range: 0–12). The total score was interpreted as follows: a total score < 3 was defined as none to low symptom load, as this threshold is used in clinical practice to define successful treatment [2], moderate symptom load was defined as 4–8 points, and scores > 9 points were regarded as high symptom load.

The Achalasia Severity Questionnaire (ASQ) was used to assess achalasia-specific health-related quality of life [17]. The ASQ consists of 10 items that examine food tolerance, dysphagia-related behavior modifications, pain, heartburn, distress, lifestyle limitation, and satisfaction. Total scores are obtained by summing up raw scores and transforming them to an interval scale ranging from 0–100, with 100 representing the lowest achalasia-related quality of life (high level of severity). Reliability and validity of the original English version of the ASQ have been demonstrated [17]. For the purpose of this study, the English version was translated to German and back-translated to English by two independent individuals to ensure translation accuracy. Cronbach’s alpha obtained in this study indicated that the translated German version is reliable (Cronbach’s alpha: .81).

### Data analysis

All analyses were conducted with R and results with a p-value < .05 were considered significant. Percentages, mean and standard deviation (M, SD) or median and median average deviation (median, MAD) were calculated as appropriate to describe the distribution of sample characteristics. Fisher’s exact test and odds ratios (OR) were applied to examine differences in distributions of depressive and generalized anxiety disorder by socio-demographic and achalasia-related characteristics. The proportion of positive screens for depressive and generalized anxiety disorder in our sample were compared to normative data of the general population in Germany. The normative data of the general population in Germany were extracted from Kocalevent et al. (2013) and Löwe et al. (2008) [15, 18]. Percentile ranks (stratified by age group and sex) of the normative samples were transformed to absolute numbers of positive screens. The absolute numbers of the normative samples were then compared to rates of positive screens in the present sample by using z-tests for independent proportion. Confidence intervals were calculated with the Clopper-Pearson Exact method for binomial proportions. Binomial logistic regression was applied to explore interactions between sex and sample origin (study sample vs. population) while controlling for age group.

Stepwise hierarchical regression was used to explore associations between achalasia-related characteristics (time since diagnosis, symptom load, achalasia-related quality of life, treatment history) and study outcomes depression and anxiety in the present sample. Socio-demographic variables (age in years, sex, income, education, partnership status) and general health status (history of mental disorder, comorbidities) were entered in model 1 as control variables and achalasia-related characteristics were entered in model 2 to test their predictive value. Missing values were assumed to be missing at random and imputed with multiple imputations by chained equations as provided by the R package mice [19]. Parameter estimates were pooled over 40 imputed datasets. As the regression models showed indication of heteroscedasticity and non-normal distribution of residuals, heteroscedasticity robust standard errors were calculated, and the dependent variables were log-transformed. Upon adding variables to the model, changes in adjusted R-squared were examined and tested using F-tests to determine increments in explained variance.

## Results

A total of 1801 patients were invited to participate in the study, of which 993 gave written informed consent and returned the questionnaire (response rate: 55.14%). Of these patients 610 (61.4%) completed the survey online and 383 (38.6%) completed the questionnaire using a paper-pencil version. No differences in relevant outcome variables or achalasia-related characteristics were shown with respect to response modality (online vs. paper-pencil). Table 1 shows the distribution of sample characteristics. The average age of participants was 52.7 years (SD = 14.4 years) and the majority of participants was in a partnership (78%). The median time that had passed since diagnosis of achalasia was 10 years (MAD = 8.90), and there was indication that the majority of participants had received treatment for achalasia (93.9%, missing responses: n = 175 [21.4%]). More than half of the participants reported at least moderate symptom severity (Eckardt score > 3, 58.3%) and at least one comorbid illness (61.9%).

**Table 1:** Participants’ sociodemographic and health-related characteristics.

		Total (N = 993)
		n (%)^a^
**Sociodemographic characteristics**		
**Age**	<25	28 (2.8)
	25–34	97 (9.8)
	35–44	135 (13.7)
	45–54	289 (29.2)
	55–64	222 (22.4)
	65–74	148 (15)
	>74	70 (7.1)
	NA	4
**Education**	≥ 12 years	462 (47.2)
	< 12 years	516 (52.8)
	NA	15
**Monthly household income**	≤ 1500 Euro	331 (34.6)
	>1500 - <4500	475 (49.6)
	≥ 4500 Euro	152 (15.9)
	NA	35
**Sex**	Female	484 (54.1)
	Male	411 (45.9)
	NA	98
**Partnership**	No	218 (22)
	Yes	774 (78)
	NA	1
**Achalasia-related characteristics**		
**Time since diagnosis of achalasia, *years***	0–5	270 (28.5)
	6–18	423 (44.7)
	>18	253 (26.7)
	NA	47
**Treatment of achalasia, *lifetime***	No	50 (6.1)
	Yes	768 (93.9)
	NA	175
**Comorbid illness, *current***	No	378 (38.1)
	Yes	613 (61.9)
	NA	2
**Symptom severity (Eckhardt score)**	0–3	402 (41.7)
	4–8	500 (51.8)
	9–12	63 (6.5)
	NA	28
**Mental health**		
**Depressive Disorder, *current*^*b*^**	No	838 (84.6)
	Yes	153 (15.4)
	NA	2
**Generalized Anxiety Disorder, *current*^*c*^**	No	889 (90)
	Yes	99 (10)
	NA	5
**Help seeking for psychological problems, *lifetime***	No	716 (72.3)
	Yes	275 (27.7)
	NA	2

Notes.

^a^ Percentages are calculated from valid case;

^b^ PHQ-9 score ≥ 10;

^c^ GAD-9 score ≥ 10

Overall, 15.4% (95% CI: 13.2–17.8) of participants screened positive for depression and 10.0% screened positive for anxiety (95% CI: 8.2–12.1). Compared to male participants, women were 1.85 (OR 95% CI: 1.24–2.77, p < .002) times more likely to screen for depression (men: 11.2%, women: 18.9%) and 2.59 (OR 95% CI: 1.55–4.46) times more likely to screen for anxiety (men: 5.6%, women: 13.3%, p < .001). Participants who reported at least moderate symptom load (Eckardt score > 3) were 3.05 (OR 95% CI: 2.00–4.78, p < .001) times more likely to screen for depression (8.0% vs. 21.0%) and 3.71 (OR 95% CI: p < .001) times more likely to screen for anxiety (4.2% vs. 14.1%), relative to participants with low symptom load.

**Fig 1:**
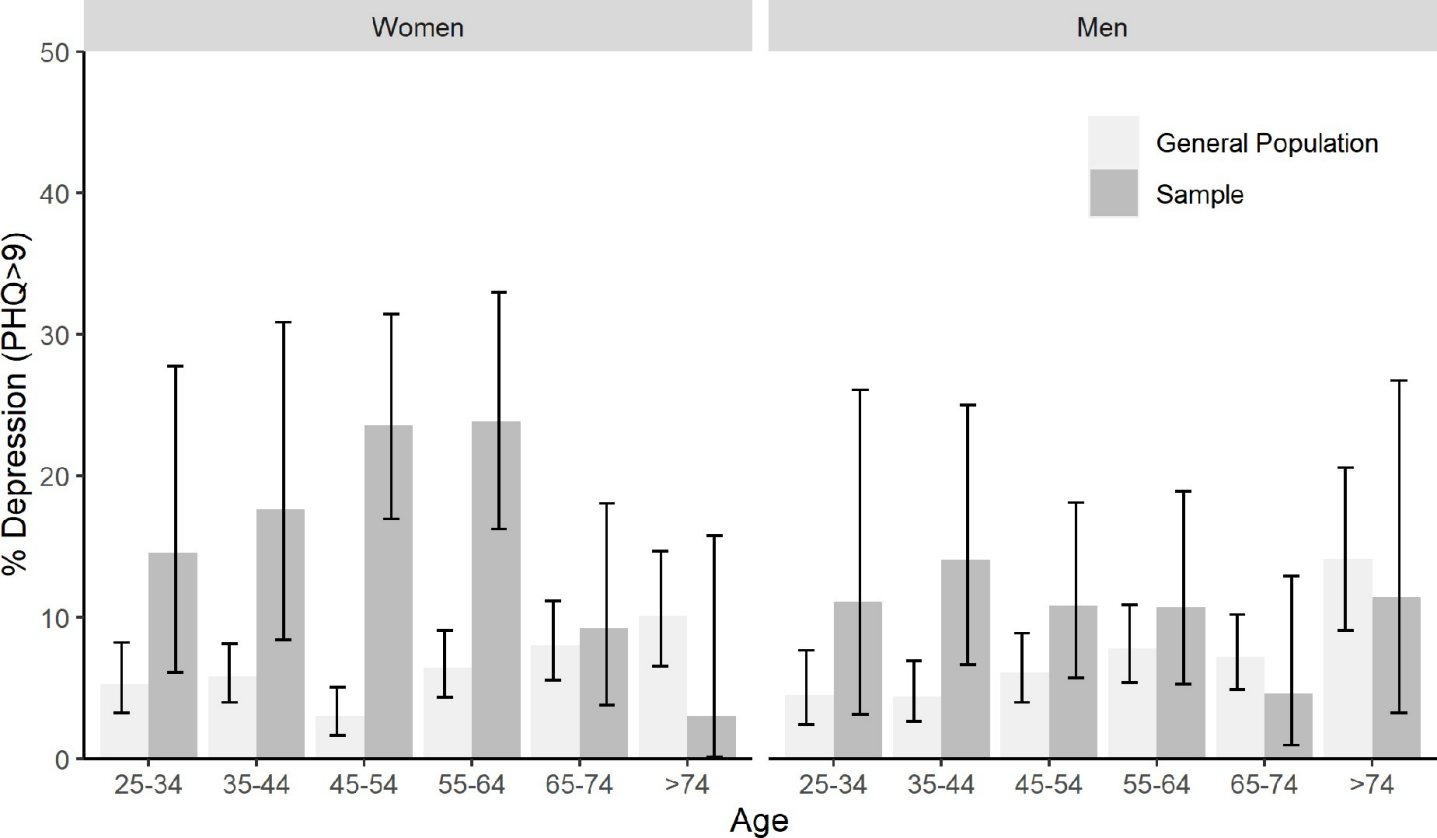
Positive screens for depression by age and sex.

**Fig 2:**
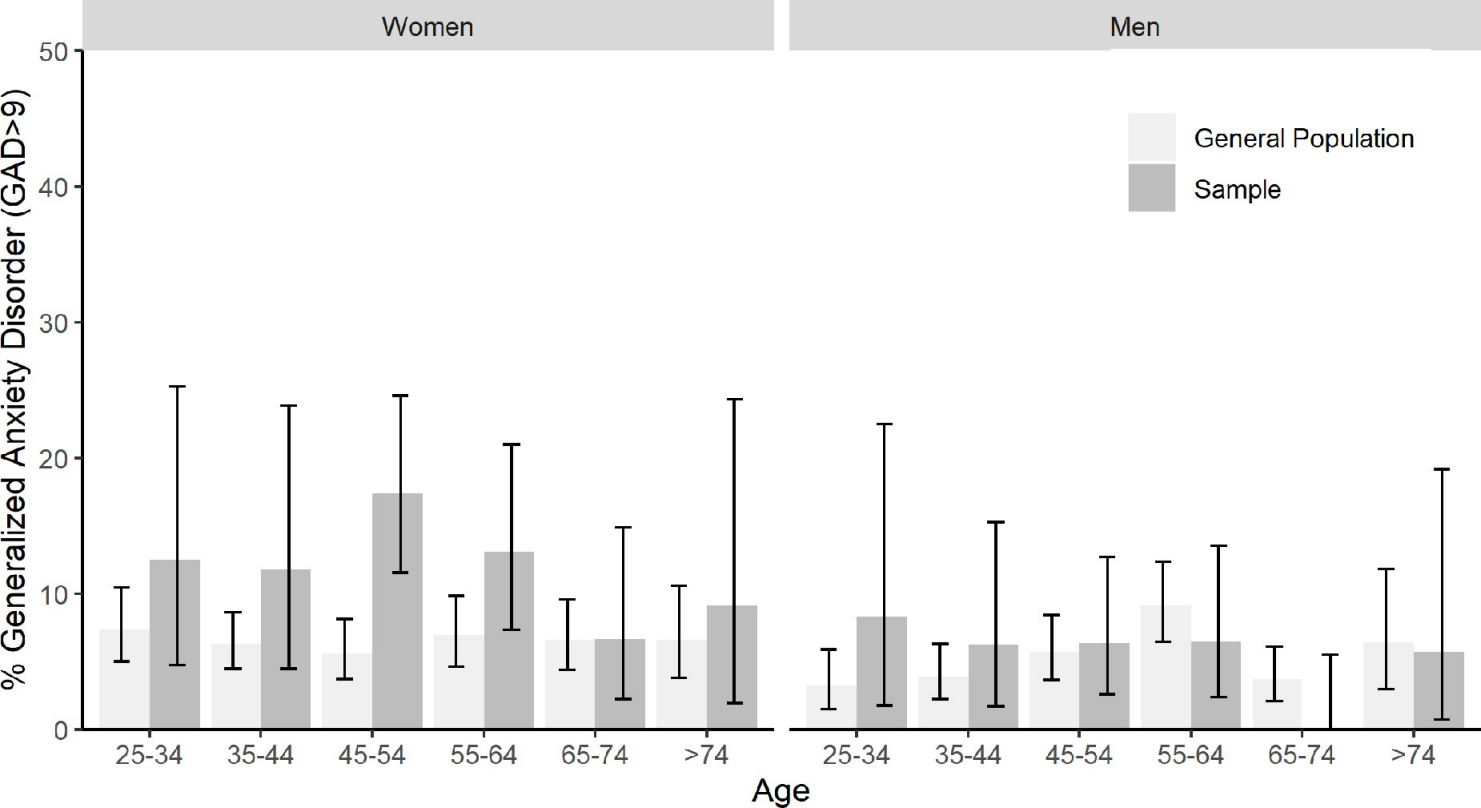
Positive screens for generalized anxiety disorder by age and sex.

Figs 1 and 2 show comparisons of screening rates of depression and anxiety, respectively, between the general population and the study sample stratified by age and sex (see also S1 and S2 Tables for raw numbers). On a descriptive level, screening rates in the study sample were elevated compared to the general population for most age and sex subgroups. With regard to depression, significant differences emerged among women in the age groups 35–44, 45–54, and 55–64. Screening rates in the study sample were 3.04 (p = .004), 7.87 (p < .001), and 3.73 (p < .001) times higher than in the general population, respectively. With regard to anxiety, screening rates were significantly elevated for women aged between 45–54, who reported 3.10 times more screens than general population controls (p < .001). We found significant interaction effects between sex and sample origin (study sample vs. general population): The difference between study sample and general population screening rates was increased for women compared to men (interaction term sex x sample origin: OR depression = 2.17 [95% CI: 1.36–3.47, p < .001], OR anxiety = 2.04 [95% CI: 1.17–3.66, p = .014]).

The results of the stepwise hierarchical regression models are presented in Table 2. As the proportion of participants with missing responses in history of treatment of achalasia was relatively large (21.4%), we decided to exclude this variable from the imputation procedure and subsequent analyses. When added to the model, achalasia-related characteristics (Step 2) significantly incremented the amount of explained variance with regard to both depressive (+10.7%, p < .001) and anxiety symptoms (+7.3%, p < .001). After controlling for covariates, achalasia-related quality of life and symptom load were significantly associated with both anxiety and depression, independent of each other. Compared to symptom load, a standardized one-unit increase in achalasia-related quality of life was related to a greater increase in depression (b = 14.9 [95% CI: 8.2–22.1] vs. b = 16.8 [95% CI: 9.8–24.2]) and anxiety scores (b = 8.6 [95% CI: 1.5–16.1] vs. b = 18.5 [95% CI: 11.1–26.5]).

**Table 2:** Regression analysis for depressive and anxiety symptoms in patients with achalasia.

	Depressive symptoms (PHQ-9)		Anxiety symptoms (GAD-7)	
	*b* (95% CI)	*b* (95% CI))	*b* (95% CI)	*b* (95% CI)
**Step 1: Covariates**	**Model 1**	**Model 2**	**Model 1**	**Model 2**
**Age in years**	-7.2 (-12.1–2)*	-0.9 (-6.2–4.7)	-7.8 (-12.8–2.6)*	-2.7 (-8-2.9)
**Sex, *female***	15.1 (3.8–27.5)*	10 (-0.2–21.3)	17.6 (5.6–30.8)*	12.6 (1.6–24.9)*
**Partnership, *yes***	-4.9 (-16.2–8)	-4 (-14.8–8.3)	6.7 (-6.1–21.2)	7.6 (-4.7–21.5)
**Income**				
**< 2000 Euro**	12.3 (0.2–25.7)*	7.2 (-3.6–19.2)	17.9 (4.9–32.5)*	13.4 (1.3–26.9)*
**2000–4500**				
**> 4500 Euro**	-6.3 (-18.7–8.1)	-3.2 (-15.4–10.7)	-4.6 (-17.7–10.5)	-1.6 (-14.7–13.4)
**Education, *≥ 12 years***	-3.7 (-13-6.7)	1.5 (-7.6–11.6)	-2.4 (-12.2–8.6)	1.9 (-8.1–12.9)
**Help-seeking for mental problems (lifetime), *yes***	68.3 (51.7–86.6)*	58.5 (43.7–74.9)*	77.7 (59.6–97.9)*	68 (51.4–86.5)*
**Comorbid illness (current), *yes***	27.9 (15–42.2)*	21.2 (9.6–34.2)*	15 (3.1–28.2)*	9.6 (-1.4–21.7)
**Step 2: Achalasia-related characteristics**				
**Time since diagnosis, *years***		-6.5 (-11—1.7)*		-5 (-9.9–0.2)
**HQoL (ASQ)**		16.8 (9.8–24.2)*		18.5 (11.1–26.5)*
**Symptom load (Eckhardt score)**		14.9 (8.2–22.1)*		8.6 (1.5–16.1)*
**R²**	0.141	0.247	0.142	0.215
**F ∆R²**	59.07	45.13	57.21	29.3

Notes. n = 993, parameter estimates pooled over 40 multiple imputations; *b* coefficient indicating percentage increase in outcome associated with one unit increase in predictor (continuous predictors were standardized: 1 unit = 1 SD); *95%CI* 95% confidence intervals based on heteroscedasticity-robust standard errors; *R²* adjusted explained variance by model; *F ∆R²* significance test of change in explained variance by predictors added to model; ASQ Achalasia Severity Questionnaire;

*p ≤ .05.

### Sensitivity analyses

To account for treatment effects, screening rates of depression and anxiety were re-estimated in the subgroup of participants who reported a history of treatment of achalasia (n = 768) and compared with population controls. Patterns of elevated screening rate in the sub-group of participants with a treatment history were largely comparable to the full sample, although women in the age group 35–44 did not show a significantly higher proportion of positive depression screens (see S3 and S4 Tables). When history of treatment of achalasia was included in the imputation, and subsequent regression models, no significant effect of history of treatment of achalasia on depressive or anxiety symptoms emerged (see S5 Table).

## Discussion

Patients with achalasia have reported reduced (disease-specific and general) health-related quality of life, but little is known regarding the psychological burden of achalasia with regard to specific mental disorders. In this study, overall screening rates of depression and anxiety were 15.4% and 10.0%, respectively. Compared to the general population, women but not men showed significantly elevated levels of depression and anxiety, with screening rates between 3.04 and 7.78 times higher than in the general population. Both more severe symptoms of achalasia and reduced achalasia-related quality of life experienced by participants were independently associated with higher levels of depression and anxiety.

Women showed larger increments in screening rates for depression and anxiety compared to population controls than men, suggesting that women may be more affected by the psychological burden associated with achalasia. Prevalence rates of most mental health disorders, including depression and anxiety, are generally higher in women than men [20]. Such differences in mental health have been linked to an interaction between biological and social factors such as gender role stereotypes and women’s social status, which can lead to exposure to different risk factors (e.g., sexual violence), inequities in access to healthcare, and maladaptive coping strategies in response to stressful life events (e.g., rumination) [21, 22]. This existing gender gap in mental health may have been aggravated by differences in psycho-social experiences of achalasia between women and men. Sex-specific or gendered differences in depressive symptoms in our sample of patients with achalasia were partly explained by the reduced achalasia-related quality of life, providing tangible support for this assumption. For example, symptoms of achalasia include abnormal bodily reactions such as dysphagia or regurgitation, and women have been shown to be more susceptible to body shame, which mediated the association between gender and depression [22]. However, sex- or gender-specific differences in anxiety symptoms in our sample could neither be explained by achalasia symptoms nor by achalasia-related quality of life, emphasizing the need for additional research on the issue of gender in achalasia. It should also be noted that men showed elevated levels of depression and anxiety on a descriptive level, but results could not be disentangled from sampling bias.

In addition, significant differences in screening rates of depression and anxiety emerged among women with achalasia compared to the general population for specific age groups (depression: age groups 35–44, 45–54, and 55–64; anxiety: age group 45–54), implying an age effect. However, when achalasia-related characteristics (symptom severity and achalasia-related quality of life) were added to the model, the impact of age on patients’ depression and anxiety scores was diminished. This suggests that the effect of achalasia-related characteristics on depression and anxiety scores in women with achalasia is greater than the age effect.

Furthermore, we found that patients with low income (< 2000 €) reported higher rates of anxiety, regardless of sex, general health status, or achalasia-related characteristics. This finding aligns with overall elevated rates of anxiety in individuals with low income [15]. Regarding symptoms of depression, low-income individuals did not report higher scores after accounting for achalasia-related factors. Due to the cross-sectional design and possible sampling bias of the current study, no causal inferences can be drawn, and this finding should be interpreted with caution. Future studies should incorporate more socioeconomic information in order to replicate and disentangle this correlation.

We further found that reduced achalasia-related quality of life was related to increased symptoms of depression and anxiety, independent of achalasia symptom severity. This result corroborates other findings suggesting that multiple factors should be considered when targeting outcomes in patients with achalasia. While treatment options such as peroral endoscopic myotomy (POEM) or Laparoscopic Heller myotomy have proven effective in reducing achalasia symptom severity, they may be less effective in improving patients’ achalasia-related quality of life in the long term [6, 23, 24]. Reduced achalasia-related quality of life could, in turn, increase the psychological burden in terms of depression and anxiety in patients, as indicated by our findings. Targeting the psychological processes underlying patient’s subjective disease experiences, such as coping strategies, psychological flexibility, or emotion regulation may improve outcomes in patients with achalasia. Well-established psychological interventions such as cognitive behavioral therapy have been shown to decrease depression and anxiety in patients with chronic illness, and more novel approaches such as acceptance and commitment therapy may be particularly suited to decrease distress resulting from chronic conditions [25, 26].

This study has several limitations. The study was based on a clinical sample of patients with achalasia recruited from a large multi-center registry with a response rate of 55.14%. Thus, generalizability of results beyond the population of individuals with comparable clinical and socio-demographic characteristics is limited. Furthermore, for some participants, data for treatment history with regard to achalasia was missing. As treatment history has been associated with well-being in patients with achalasia, this may have biased results. However, studies indicate that mental health outcomes following treatment for achalasia are driven mainly by changes in symptom severity, reducing the relevance of treatment history as an explanatory variable [7, 27, 28]. In this sample, patients in clinical remission (i.e., Eckardt score < 3) and with at least moderate symptom load were relatively evenly distributed. Moreover, sensitivity analyses suggest that treatment history was not associated with outcomes in this study. We used the PHQ-9 and GAD-7 in this study, which have shown favorable sensitivity and specificity in detecting depression and anxiety, respectively [14, 15]. However, these instruments are not suited to establish a clinical diagnosis. Thus, rates of depression and anxiety estimated by these instruments may not reflect the true prevalence of disorders and should be regarded as preliminary estimates.

## Conclusions

The findings of this study point to sex-specific or gendered experiences of the psychological burden of achalasia. Women with achalasia were more likely to screen for depression and anxiety compared to population controls than men with achalasia. Both more severe symptoms of achalasia and reduced achalasia-related quality of life experienced by participants were independently associated with higher levels of depression and anxiety. These findings indicate that addressing psychological factors associated with achalasia-related quality of life in addition to therapeutic interventions aiming at symptom reductions could be beneficial for patients with achalasia. Further research on the issue of gender in achalasia is needed to investigate underlying mechanisms and provide targeted support.

## Supporting information

S1 Table Proportion of positive screens for depressive disorders (PHQ-9 score ≥ 10): Full sample. (DOCX)

S2 Table Proportion of positive screens for generalized anxiety disorders (GAD-7 score ≥ 10): Full sample. (DOCX)

S3 Table Proportion of positive screens for depressive disorders (PHQ-9 score ≥ 10): Treatment subgroup. (DOCX)

S4 Table Proportion of positive screens for generalized anxiety disorders (GAD-7 score ≥ 10): Treatment subgroup. (DOCX)

S5 Table Sensitivity analyses with treatment included. (DOCX)
