# Supplementary material for: Psychological burden of achalasia: Patients’ screening rates of depression and anxiety and sex differences
Source: PLoS One. 2023 May 11;18(5):e0285684. doi: 10.1371/journal.pone.0285684 (PMC10174570; doi:10.1371/journal.pone.0285684)
Supplement: S1 Table — (DOCX) [file pone.0285684.s001.docx]

**Supplementary Material**

S1 Table. Proportion of positive screens for depressive disorders (PHQ-9 score ≥ 10): Full sample.

|  | Women | | Men | |
| --- | --- | --- | --- | --- |
| Age group | Sample in % (95% CI) [n/N] | General population^a^ in % [n/N] | Sample in % [n/N] | General population^a^ in % [n/N] |
| 25-34 | 14.6 (6.1-27.8)[7/48] | 5.3 (3.2-8.2)[18.6/351] | 11.1 (3.1-26.1)[4/36] | 4.5 (2.4-7.6)[12.6/279] |
| 35-44 | 17.6 (8.4-30.9)[9/51]* | 5.8 (4-8.1)[31.4/542]* | 14.1 (6.6-25)[9/64] | 4.4 (2.6-6.9)[17.4/396] |
| 45-54 | 23.6 (16.9-31.4)[34/144]* | 3 (1.6-5)[13.7/457]* | 10.8 (5.7-18.1)[12/111] | 6.1 (4-8.9)[25.3/414] |
| 55-64 | 23.9 (16.2-33)[26/109]* | 6.4 (4.3-9.1)[28.5/446]* | 10.8 (5.3-18.9)[10/93] | 7.8 (5.4-10.9)[31/398] |
| 65-74 | 9.2 (3.8-18.1)[7/76] | 8 (5.5-11.1)[31.6/395] | 4.6 (1-12.9)[3/65] | 7.2 (4.9-10.2)[28.6/397] |
| >74 | 3 (0.1-15.8)[1/33] | 10.1 (6.6-14.7)[23.8/236] | 11.4 (3.2-26.7)[4/35] | 14.1 (9.1-20.6)[22/156] |

Notes. Total size study sample N =865, only participants with valid responses included; ^a^ Prevalence estimate obtained from Kocalevent et al. [20]; *significant difference between study sample and general population with Bonferroni-Holm adjusted p-level, number of comparison: n=12)
