# Supplementary material for: Psychological burden of achalasia: Patients’ screening rates of depression and anxiety and sex differences
Source: PLoS One. 2023 May 11;18(5):e0285684. doi: 10.1371/journal.pone.0285684 (PMC10174570; doi:10.1371/journal.pone.0285684)
Supplement: S2 Table — (DOCX) [file pone.0285684.s002.docx]

S2 Table. Proportion of positive screens for generalized anxiety disorders (GAD-7 score ≥ 10): Full sample.

|  | Women | | Men | |
| --- | --- | --- | --- | --- |
| Age group | Sample in % (95% CI) [n/N] | General population^a^ in % [n/N] | Sample in % [n/N] | General population^a^ in % [n/N] |
| 25-34 | 12.5 (4.7-25.2)[6/48] | 7.4 (5-10.5)[28.6/387] | 8.3 (1.8-22.5)[3/36] | 3.2 (1.5-5.9)[9.5/297] |
| 35-44 | 11.8 (4.4-23.9)[6/51] | 6.3 (4.4-8.6)[35.7/566] | 6.2 (1.7-15.2)[4/64] | 3.9 (2.2-6.3)[15.5/398] |
| 45-54 | 17.4 (11.6-24.6)[25/144]* | 5.6 (3.7-8.1)[25.6/457]* | 6.4 (2.6-12.7)[7/110] | 5.7 (3.7-8.4)[23.1/406] |
| 55-64 | 13.1 (7.3-21)[14/107] | 6.9 (4.6-9.8)[28/406] | 6.5 (2.4-13.5)[6/93] | 9.1 (6.5-12.4)[36.4/400] |
| 65-74 | 6.7 (2.2-14.9)[5/75] | 6.6 (4.3-9.5)[25.6/388] | 0 (0-5.5)[0/65] | 3.7 (2.1-6.1)[14.6/394] |
| >74 | 9.1 (1.9-24.3)[3/33] | 6.6 (3.8-10.6)[15.4/234] | 5.7 (0.7-19.2)[2/35] | 6.4 (3-11.8)[9/141] |

Notes. Total size study sample N =861, only participants with valid responses included; ^a^ Prevalence estimate obtained from Löwe et al. [13]; *significant difference between study sample and general population with Bonferroni-Holm adjusted p-level, number of comparison: n=8) , age groups 25-34 and 35-44 not tested due to small subgroup size in study sample (n≤15)
