# Supplementary material for: Psychological burden of achalasia: Patients’ screening rates of depression and anxiety and sex differences
Source: PLoS One. 2023 May 11;18(5):e0285684. doi: 10.1371/journal.pone.0285684 (PMC10174570; doi:10.1371/journal.pone.0285684)
Supplement: S3 Table — (DOCX) [file pone.0285684.s003.docx]

S3 Table. Proportion of positive screens for depressive disorders (PHQ-9 score ≥ 10): Treatment subgroup.

|  | Women | | Men | |
| --- | --- | --- | --- | --- |
| Age group | Sample in % (95% CI) [n/N] | General population^a^ in % [n/N] | Sample in % [n/N] | General population^a^ in % [n/N] |
| 25-34 | 12.8 (4.3-27.4)[5/39] | 5.3 (3.2-8.2)[18.6/351] | 12.5 (3.5-29)[4/32] | 4.5 (2.4-7.6)[12.6/279] |
| 35-44 | 17.5 (7.3-32.8)[7/40] | 5.8 (4-8.1)[31.4/542] | 15.1 (6.7-27.6)[8/53] | 4.4 (2.6-6.9)[17.4/396] |
| 45-54 | 22.1 (14.9-30.9)[25/113]* | 3 (1.6-5)[13.7/457]* | 11 (5.4-19.3)[10/91] | 6.1 (4-8.9)[25.3/414] |
| 55-64 | 22.4 (13.6-33.4)[17/76]* | 6.4 (4.3-9.1)[28.5/446]* | 11 (4.9-20.5)[8/73] | 7.8 (5.4-10.9)[31/398] |
| 65-74 | 10.2 (3.4-22.2)[5/49] | 8 (5.5-11.1)[31.6/395] | 2.1 (0.1-11.3)[1/47] | 7.2 (4.9-10.2)[28.6/397] |
| >74 | 5.3 (0.1-26)[1/19] | 10.1 (6.6-14.7)[23.8/236] | 9.1 (1.1-29.2)[2/22] | 14.1 (9.1-20.6)[22/156] |

Notes. Total size study sample N = 654, only participants with treatment history of achalasia and valid responses included; ^a^ Prevalence estimate obtained from Kocalevent et al. [20]; *significant difference between study sample and general population with Bonferroni-Holm adjusted p-level, number of comparison: n=12).
