# Supplementary material for: Psychological burden of achalasia: Patients’ screening rates of depression and anxiety and sex differences
Source: PLoS One. 2023 May 11;18(5):e0285684. doi: 10.1371/journal.pone.0285684 (PMC10174570; doi:10.1371/journal.pone.0285684)
Supplement: S4 Table — (DOCX) [file pone.0285684.s004.docx]

S4 Table. Proportion of positive screens for generalized anxiety disorders (GAD-7 score ≥ 10): Treatment subgroup.

|  | Women | | Men | |
| --- | --- | --- | --- | --- |
| Age group | Sample in % (95% CI) [n/N] | General population^a^ in % [n/N] | Sample in % [n/N] | General population^a^ in % [n/N] |
| 25-34 | 7.7 (1.6-20.9)[3/39] | 7.4 (5-10.5)[28.6/387] | 9.4 (2-25)[3/32] | 3.2 (1.5-5.9)[9.5/297] |
| 35-44 | 12.5 (4.2-26.8)[5/40] | 6.3 (4.4-8.6)[35.7/566] | 7.5 (2.1-18.2)[4/53] | 3.9 (2.2-6.3)[15.5/398] |
| 45-54 | 16.8 (10.4-25)[19/113]* | 5.6 (3.7-8.1)[25.6/457]* | 5.6 (1.8-12.5)[5/90] | 5.7 (3.7-8.4)[23.1/406] |
| 55-64 | 12 (5.6-21.6)[9/75] | 6.9 (4.6-9.8)[28/406] | 6.8 (2.3-15.3)[5/73] | 9.1 (6.5-12.4)[36.4/400] |
| 65-74 | 8.3 (2.3-20)[4/48] | 6.6 (4.3-9.5)[25.6/388] | 0 (0-7.5)[0/47] | 3.7 (2.1-6.1)[14.6/394] |
| >74 | 10.5 (1.3-33.1)[2/19] | 6.6 (3.8-10.6)[15.4/234] | 4.5 (0.1-22.8)[1/22] | 6.4 (3-11.8)[9/141] |

Notes. Total size study sample N = 651, only participants with treatment history of achalasia and valid responses included; ^a^ Prevalence estimate obtained from Löwe et al. [13]; *significant difference between study sample and general population with Bonferroni-Holm adjusted p-level, number of comparison: n=12).
