# Supplementary material for: Psychological burden of achalasia: Patients’ screening rates of depression and anxiety and sex differences
Source: PLoS One. 2023 May 11;18(5):e0285684. doi: 10.1371/journal.pone.0285684 (PMC10174570; doi:10.1371/journal.pone.0285684)
Supplement: S5 Table — (DOCX) [file pone.0285684.s005.docx]

S5 Table. Sensitivity analyses with treatment included.

|  | Depressive symptom (PHQ-9) | | Anxiety symptoms (GAD-7) | |
| --- | --- | --- | --- | --- |
|  | *b* (95% BcaCI) | *b* (95% BcaCI)) | *b* (95% CI) | *b* (95% CI) |
| Step 1: Covariates | **Model 1** | **Model 2** | **Model 1** | **Model 2** |
| Age | -8.1 (-13.3--2.6)* | -1.5 (-7.3-4.6) | -8 (-13.4--2.3)* | -2.3 (-8.1-4) |
| Gender, *female* | 12.2 (-0.2-26.1) | 7.7 (-3.4-20.2) | 17.6 (4.4-32.5)* | 13.5 (1.3-27.3)* |
| Partnership, *yes* | -2.8 (-16.1-12.6) | -0.7 (-13.4-14) | 16.1 (0.1-34.7)* | 18.2 (2.8-36)* |
| Income |  |  |  |  |
| < 2000 Euro | 14.3 (0.7-29.7)* | 9.9 (-2.3-23.6) | 18.1 (3.8-34.4)* | 14.3 (1-29.4)* |
| 2000 - 4500 |  |  |  |  |
| > 4500 Euro | -4.8 (-18.2-10.9) | -1.8 (-15-13.5) | -4.2 (-18.1-12.2) | -1.4 (-15.6-15.1) |
| Education, *≥ 12 years* | -0.4 (-11-11.4) | 5.4 (-5-17) | 4.4 (-7.2-17.5) | 9.4 (-2.4-22.5) |
| Help-seeking for mental problems (lifetime), *yes* | 72.9 (54.3-93.8)* | 63.7 (46.8-82.4)* | 79.6 (59.8-101.9)* | 71.3 (52.9-91.9)* |
| Comorbid illness (current), *yes* | 35.8 (21.4-52)* | 26.8 (13.8-41.4)* | 19.2 (6-34.1)* | 12.4 (0.2-26)* |
| Step 3: Achalasia-related characteristics |  |  |  |  |
| Time since diagnosis, *years* |  | -5.9 (-10.9--0.7)* |  | -5.6 (-10.9-0) |
| HQoL (ASQ) |  | 16.7 (9.2-24.8)* |  | 16.1 (8.2-24.6)* |
| Symptom load (Eckhardt score) |  | 16.2 (8.7-24.2)* |  | 11.4 (3.4-20)* |
| Treatment (lifetime), *yes* |  | 4.3 (-15.2-28.3) |  | 6.5 (-11.8-28.5) |
| R² | 0.153 | 0.262 | 0.147 | 0.211 |
| F ∆R² |  | 39.0 |  | 24.9 |

Notes. n = 993, parameter estimates pooled over 40 multiple imputations; *b* coefficient indicating percentage increase in outcome associated with one unit increase in predictor (continuous predictors were standardized: 1 unit=1 SD); *95%CI* 95% confidence intervals based on heteroscedasticity-robust standard errors; *R²* adjusted explained variance by model*; F ∆R²* significance test of change in explained variance by predictors added to model; ASQ Achalasia Severity Questionnaire; **p* ≤ .05.
